# Cancer: An Approach from the Nutritional Side

**Published:** 1915-07

**Authors:** S. W. Little

**Affiliations:** Rochester, N. Y.


### Cancer: An Approach From the Nutritional Side.

S. W. LITTLE, M. D., Rochester, N. Y.

It must be admitted that cancer is a disorder of metabolism. That is to say, cancer consists of living cells; every living cell must take in food from outside itself; the only ultimate source of food therefore for a cancer cell is material taken in from outside the organism in which the cancer exists. A man who has a cancer then is feeding that cancer with what he takes into his stomach or his lungs. Nor will it make any difference if one should grant that the cancer started by taking in some special food or drink or air, for after the disease is recognized and treated, certainly the patient takes in nothing peculiar and still the thing grows. Nor will it discredit the opening statement to invoke the aid of bacteria or other such organisms. Grant such an origin of cancer (the writer does not) still the cancer grows from food taken into the host. And by the way, a cancer is no more remarkable a phenomenon biologically than a lipoma. No one has hinted at germs seriously in the case of lipomata. It might clear up the cancer matter if we could prove the cause of some of the simpler new growths such as lipomata. One does not dash at logarithms before he can add. Yet that is exactly what we are doing (the writer included) in the case of cancer. The clinical importance of cancer is our excuse if not our reason. The writer offers a further excuse in his own case because if his idea is correct (size of "if" granted) he offers a possible solution of the mystery of certain so-called "new growths."

Cancer then is a disorder of metabolism. As Vaughan points out our raw material, meat, vegetables, etc., is first roughly worked over in the gastro-intestinal tract for the whole organism. But this is only the beginning. Proteids and carbohydrates in general must go next to the liver to be metabolized further. Fats in general dodge the liver. All three then of necessity go through the lungs (nor is it at all proven so far as the writer knows that the red blood cells are the only elements that pick up material there) and then to the entire body. But by no means is that all. Each kind of cell after it gets its food must then work it over in its own way within its own cell body, and furthermore before many highly-specialized cells can even begin that process some further unknown treatment of the food is apparently needed.

All this merely states in another way that a very important and not sufficiently emphasized distinction exists between a unicellular organism and any single cell of a multicellular organism. To be sure both are biologically alike in that they must take in food and must give off waste matter. But a cell living by itself lives without reference to any other cell; it is self-sufficient. A cell which is merely one of many cells in one organism is less independent than one citizen in a republic. It is absolutely dependent for its life upon its associates and has surrendered many vital functions to its associates which the single cell of a unicellular organism does for itself. The latter moves, absorbs, feels, digests, excretes, reproduces by itself; whereas in the other case a given cell may be able to do only a part of one of these functions, relying upon its fellows to do the rest. For example, a muscle cell in one's biceps cannot even move of itself; it must have the order to move, i. e. the stimulus, brought to it by other cells. It cannot alone even obtain its food, much less prepare it. These things are done by other cells. All this of course is specialization in the interest of efficiency. And right here is the very important distinction between a unicellular organism and a single cell of a multicellular organism. The latter functionates not for itself alone, indeed scarcely at all for itself, but for the entire organism. A red blood cell certainly does not carry its load of oxygen for itself, a cell in the stomach does not manufacture HCl for itself alone and so on. No single normal highly specialized cell in the human body can do the variety of things an amoeba does. In fact no such cell can even live apart from its fellows, by living being understood the essential functional ability by itself to take in and digest raw material, to grow, to excrete waste and to reproduce itself. When such a phenomenon exists in the human organisms we rightly call it pathological and in the case of a certain class of cell we call it cancer. That is to say, a cancer cell is a cell of this class which, ceasing to perform a special work for the good of the organism and regaining the ability to perform the elemental functions originally belonging to all cells, no longer functionates for the common good but for itself alone. It eats, grows, excretes, and is able to reproduce its kind; it is practically a unicellular organism. It is an outlaw in the human body politic. Like human outlaws, cancer cells if numerous or powerful enough destroy the organism they infest. And like human outlaws cancer cells can theoretically be rendered harmless in only one of three ways. By killing, by imprisoning or by reformation. Our only certain way at present is to kill by removal and destruction. Various attempts, unsuccessful for the most part thus far, have been made to kill by poisoning them and by burning them. They are occasionally more or less successfully imprisoned for longer or shorter terms by the efforts of the law-abiding cells. It is conceivable that we may ultimately learn the secret and aid in this way.

Reformation may seem at present visionary but may be by no means really so judging from one or two of the writer's experiences.

The idea is this: may we not either kill these cells by starving them or possibly even reform them by allowing them **to get only the kind of food suitable for the specialized cells they once were?** Or at all events may we not furnish large amounts of this specialized food and thereby greatly improve the condition of their fellow cells which have remained true to type, thus greatly strengthening the hand of the whole organism in its fight with the outlaws? It is the author's idea that these parasites start their career only when such specialized food fails, because they thrive on less thoroughly metabolized foods which the law abiding cells cannot manage; and he proposes to kill them or to reform them by supplying only the normal specialized food. Just as one would either kill a savage or render him gastronomically civilized if one should suddenly and persistently feed him such fearful and wonderful diet as civilized people ingest with a fair degree of impunity.

For the whole argument the reader is referred to former articles in the Boston Medical and Surgical Journal (Jan., Feb. and Oct., 1914) and in the N. Y. S. Journal of Medicine (April 1915). The theory rests on the reasoned necessity of the secretion of certain ductless glands for specialized cell nutrition. A single example will explain the idea. The Islands of Langerhans are absolutely essential if certain cells are to obtain their necessary supply of carbohydrate food. In diabetes of this type there is plenty of glucose in the blood but without the islands it is largely of no avail. That is, for certain specialized cells, these islands are essential in the process of preparing a raw starch granule so that it will be available for food. It is the final stage in rendering raw material fit for food in the briefly sketched metabolic outline given above. Now for such a highly differentiated cell in its early embryonic stage it is evident that no such complicated metabolic machinery is necessary, in fact does not exist. The ductless glands do not appear until differentiation has begun. Like all other simpler cells the metabolic processes of embryonic cells are simpler.

Imagine a peptic cell in the stomach which (see former articles) has undergone a retrograde metamorphosis until it is no longer a highly differentiated cell but a much simpler embryonic peptic cell. Imagine that some ductless gland essential to the nutrition of a normal adult peptic cell has failed more or less from age, disease, hard usage or the like. The food supply is therefore becoming more and more difficult for the normal cell to manage while the retrograde embryonic cell thrives on the more simply prepared food. The result is plain. The normal cells succumb and the embryonic cells thrive. We have a cancer.

Essentially then the theory is that cancer consists of certain highly specialized cells which are in or have reverted to an embryonic stage and that these cells reproduce at the expense of the organism if certain ductless glands are hypofunctionating.

The only present successful treatment for such a growth consists in absolute destruction of the embryonic cells. The writer is trying to check or even to cure cancer by removing one assumed element necessary to its existence—hypofunction of certain ductless glands. The argument has been gone over in the articles referred to and cannot be briefly summarized here. The tentative conclusion reached is that for cancer of ectodermic origin, hypopituitarism is an essential; for cancer of mesodermic origin, hyposecretion of the suprarenal cortex and for endodermic cancer, hypofunction of the islands of Langerhans. It must not be understood that such hypofunction is the only factor in the production of cancer. It is merely an essential factor.

The theoretical treatment consists in correcting if possible such hypofunction. The results, aptly called tantalizing by Prof. Adami in a personal letter, are also most interesting and in a few cases most gratifying. In three cases of epitheliomata we have actually seen the growth lessen in size. In one of them we have seen it disappear; reappear on stopping medication and again disappear under medication.

We have had least success with endodermic cancer as might have been expected, for two reasons: The technical difficulty of getting a preparation of the islands of Langerhans alone and our inability thus far to devise a means for giving any preparation of the islands in a way even approximating the natural way. Remember the secretion whatever it is gets into the circulation gradually and in minute amounts at a time by way of the thoracic duct, thereby dodging the liver. We can use what we have only by mouth and in relatively sudden and enormous doses. Even this probably goes into the blood stream and therefore to the liver first. Incidentally the writer believes this fact explains in a measure the disappointing results in diabetes of pancreatic therapy.

As to dosage the writer uses from six to twenty grains daily of a reliable dry extract of the whole pituitary gland (Burrough, Welcome & Co.) from six to sixteen grains of dry suprarenal cortex extract and from two to four ounces of a fluid extract of the islands of Langerhans. The last two are not as yet on the market but have been made for the writer by the G. W. Carnrick Co.

(The writer's case histories would more than double the length of this already too-long paper. He claims no cures although some cases are apparently well. Many have died. All were inoperable for one cause or another and inasmuch as complete surgical removal is still our only certain cure, it is unfair to advise anything else in an operable case. In an inoperable case any treatment that offiers a chance is not only justifiable but imperative.)

109 Plymouth Avenue.

---

**Injuries From Aviators' Darts.** Grützner, Münch. Med. Woch., Feb. 8, 1915, estimates the striking force of a dart dropped from a height of 1500 meters at 3.428 kilogrammeters, of an infantry bullet weighing 10 grams at about half this force. The latter, shot with a muzzle velocity of 885 meters would have an impact of 8.55 kilogram-meters. Darts dropped from the usual height of 1500-2000 meters cannot penetrate bone but will pass through soft parts doing extensive damage. An artillerist, was struck in the shoulder, there being a small entrance wound at the anterior border of the clavicle. Death from collapse occurred in 24 hours. There were extravasations of blood in the left pleural and peritoneal cavities, four penetrations of mesentery, two of stomach and 12 of small intestine.

---

**Test for Urine in Preliminary Stages of Diabetes.** Bergell, Deutsche Med. Woch., No. 51, 1914, describes the following based on the observation that diabetic urine when alkalinized, dissolves more copper sulphate than normal. The urine is diluted to a specific gravity of 1012. To 20 C.C., is added 7 C.C. of 15% sodium hydroxid and the mixture is shaken once. Add 3 C.C. copper sulphate solution. Shake thoroughly. Normal urine is scarcely colored. Diabetic urine shows a deep blue color. If, in a mild case of diabetes, the urine is rendered sugar-free by diet, the color test persists for several days. Descendants of diabetics show the color test in 60% of adults, 80% of children but the test may be rendered negative by diet, after a few days. The rationale of the test is not explained.

---

**Rapid Sterilization of Water for Military Purposes.** Mendez mentions in Revista Medica de Sevilla, G. Lambert-Forment's formula. Potassium permanganate 0.05, Manganese dioxid 0.05, Powdered talc 0.37, Calcium carbonate 0.02 are added to 1 liter of water. After ten minutes, the excess of permanganate is neutralized by 0.06 Sodium hyposulphite with 0.44 talc and the liquid is decanted and filtered.

